# Genotypes of human papilloma virus in Sudanese women with cervical pathology

**DOI:** 10.1186/1750-9378-5-26

**Published:** 2010-12-30

**Authors:** Magdi M Salih, Mohamed El Safi, Keith Hart, Khater Tobi, Ishag Adam

**Affiliations:** 1 Department of Histopathology and Cytology, Faculty of Medical Laboratory Sciences, University of Khartoum, Sudan; 2 Department of Pathology, Al Ribat National University, Sudan; 3 Central Biotechnology Service, Henry Wellcome Building, School of Medicine, Cardiff University, Cardiff, UK; 4 Department of Obstetrics and Gynecology, Omdurman Military Hospital, Sudan; 5 Department of Obstetrics and Gynecology, Faculty of Medicine, University of Khartoum, Khartoum, Sudan

## Abstract

**Background:**

Knowledge of the distribution of human papillomavirus (HPV) genotypes among women with cervical lesion and in invasive cervical cancer is crucial to guide the introduction of prophylactic vaccines. There is no published data concerning HPV and cervical abnormalities in Sudan. This study aimed to define the prevalence of HPV and its subtypes in the cervical smears of women presenting with gynecological complains at Omdurman Military Hospital, Sudan.

During the period between March 2003 and April 2004, 135 cervical smears collected from these women, were screened using cytological techniques, and analysed by PCR for (beta)-globin and HPV DNA using gel electrophoresis and ELISA.

**Results:**

Of these 135 smears, there were 94 (69.3%) negative, 22 (16.3%) positive for inflammation, 12(8.9) mild dyskaryosis, 5 (3.7) moderate dyskaryosis and 2 (1.8) severe dyskaryosis. There were 60.7% ß. globin positive samples for HPV indicating DNA integrity. HPV DNA was identified in three samples (2.2%) by gel electrophoresis and. was positive in four samples (2.9%) as single and multiple infections by PCR-ELISA. The high risk HPV types 16 and 58 were identified in one sample as a mixed infection. The low risk HPV types 40 and 42 were also found as a mixed infection in another patient. HPV types 58 and 42 were identified in the other two patients.

**Conclusion:**

HPV type distribution in Sudan appears to differ from that in other countries. The HPV genotypes identified were not associated with cancer.

## Background

Human papilloma virus (HPV) is a cause of cervical cancer and *condylomata acuminata *[1]. Many sexually active populations in many parts of the world have been infected with HPV during their lifetime [2], most HPV-infected individuals eliminate the virus without developing clinical symptoms [3]. Nearly 100 HPV types have been molecularly identified and about 40 of these can infect the ano-genital tract [4]. On the basis of their oncogenic potential, most of these genital HPV types have been classified as high or low risk for causing cervical cancer. The high-risk types, especially HPV 16 and 18 are implicated in the development of cervical intraepithelial neoplasia and cervical carcinoma. The low-risk types (HPV 6, 11, 40, 42, 43, 44, 54, 61, 70, 72, 81, and CP6108) can cause mild cervical dysplasia but are rarely associated with severe cervical dysplasia or cervical carcinoma [5,6] Women found to be positive for high-risk HPV were at increased risk of developing CIN3 than those with a negative HPV test [7]. However infection by HPV does not always lead to malignant and pre-malignant change development and populations may carry HPV types that are frequently associated with cancer, and not develop invasive lesions [8]. Thus, the prevalence of HPV in a population of people is invariably greater than the number of women who develop cervical lesions. HPV type distribution and cervical cancer has been studied in other African countries [3,9]. However, there is as yet no published data from The Sudan. Thus, in the present study GP5+/GP6+-PCR EIA [*] was used to identify the prevalence of HPV genotypes among women attending the Gynaecology clinic in Omdurman Military Hospital, Sudan.

## Methods

During the period of February 2003 through May 2004, a cross-sectional study was conducted among, women presenting to the gynecological clinic with symptoms of vaginal discharge, inter-menstrual bleeding, and post-coital bleeding were approached to participate in the study. After informed consent, questionnaires were conducted to gather socio-demographic, medical and gynecological characteristics. Then cervical smears were collected using the standard Szalay cyto-spatula. Cells were initially used to prepare a Pap smear and the remaining cells on the spatula were eluted in Tris HCl buffer PH 8.0. A cell pellet was obtained by centrifugation, re-suspended in 2 ml Tris HCl buffer pH 8.0 and frozen at -20°C until HPV analysis using PCR-EIA in Cardiff, United Kingdom. Cytology and HPV DNA detection Pap smears were prepared with Papanicolaou stain, screened by cyto -screener and positive results revised and confirmed. To target DNA, samples were pre-screened by polymerase chain reaction (PCR) using ß-globin specific primers as described previously [10]. Human papilloma virus infections were analyzed by general primer GP5+/6+ mediated PCR enzyme immunoassay (EIA) as described by Jacobs et al [10] that its used to detect a broad spectrum of human papilloma virus (HPV) genotypes including the high-risk groups (HPV-16,18,31,33,35,39,45,51,52,56,58,59,66, 68 and 73) and low risk groups (HPV-6,11,40,42,43 and 44). Moreover, amplification products were analyzed for individual HPV subtypes.

## Results

The mean age of the women was 33.18 years (range 17- 60 years). Cervical smears had normal cytology in 94 (69.6%) out of 135 patients. The rest of the patients had 22(16.3) inflammatory changes 12(8.9) mild dyskaryosis, 5 (3.6) moderate dyskaryosis and 2 (1.5) severe dyskaryosis. Human DNA was detected in 82 (60.7%) out of 135 samples using ß globin PCR. Three samples (2.3%) were positive for HPV DNA as determined by gel electrophoresis; however these samples were not reactive with ELISA for HR and LR groups, suggesting novel HPV subtypes. The cytological findings of these two samples were reported as moderate dyskaryosis and severe dyskaryosis. Nine samples had a reactive result with HPV DNA using ELISA method. Three samples showed reactive result with HPV 58, these were found in samples that were reported as moderate dyskaryosis and one with mild dyskaryosis. Another two samples showed reactive result with HPV types 16 and 42, the cytological result of these were, inflammation and normal cytology respectively. The other four samples were reactive with ELISA and gel electrophoresis for HPV DNA. Those patients showed infection with general high risk (HR) cocktails group and general low risk (LR) cocktails group, table 1. After the sub typing of these two groups, two mixed (low and high risk group) infections were found. Type 16 and 58 were identified in one sample that is reported cytologically as moderate dyskaryosis, and type 40 and 42 in another single sample with negative cytological result. The most common high risk infection in our study group was type 58. And in the low risk the most common was type 42, Table 2.

**Table 1:** cervical pathology and HPV prevalence in Sudanese women

*Cytology Result*	*HPV DNA HR*		*HPV DNA LR*		*Total*
		
	Positive	Negative	Positive	Negative	
Negative	0(00%)	94(100%)	3(3.2%)	91(96.8%)	94(69.3%)
Inflammation	1(4.5%)	21(95.5%)	0 (00%)	22(100%)	22 (16.3)
Mild dyskaryosis	1(8.4%)	11 (91.6)	0 (00%)	12(100%)	12(8.9)
Moderate dyskaryosis	4 (80%)	1 (20%)	0(00%)	5 (100%)	5(3.7)
Severe dyskaryosis	0(00%)	2(100%)	0(00%)	2(100%)	2 (1.8)

**Total**	6	129	3	132	135

**Key **HR (High risk types), LR (Low risk types

**Table 2:** Cervical pathology and HPV genotypes

*HPV Types*	*Negative*	*Infection*	*mild*	*Moderate*	*Severe*	*Total*
	Infection with single HPV					
HPV 58				1		1
HPV 16		1				1
HPV 58			1	1		2
HPV 42	1					1
	Infection with mixed HPV					
HPV 16				1		1
HPV 58				1		1
HPV 40		1				1
HPV 42		1				1
	HPV DNA identified with Gel					
HPV X				2	1	3

Total	1	3	1	6	1	12

Cytological results/HPV Genotypes

## Discussion

This is the first study we are aware of investigating HPV infection among Sudanese women and one of the few studies that used the PCR-EIA technique (general primer (GP5+/GP6+) and ELISA) in Sub-Saharan Africa. Previously Ibrahim and his colleagues failed to isolate HPV DNA in a small number of oral cancer tissue samples using classical PCR techniques in Sudanese patients [11]. GP5+/bio-GP6+ PCR-EIA was the method used in the current study, which has a high sensitivity and specificity for HPV/HPV type detection. In this study, 60.7% of samples were ß globin positive, and the rest were non reactive. Perhaps the cold chain was interrupted somewhere during samples transfer. Only three samples were classified as HPV X that is HPV/DNA positive with gel electrophoresis and genotype negative with ELISA. There may be some unidentified HPV types or the types of HPV are not within the probe cocktail of the 15 high risks or the 6 low risk group. A similar result was obtained in other African countries e.g. Mozambique [12] in which HPV specific types could not be identified in 19% of the HPV positive cases; they related this failure to the limited number of HPV specific probes used. In Uganda a similar result was obtained where they identified and sequenced one other variant of HPV 16 called HPV 16 AF1 sequence [13].

HPV 58 was the most common type in the current study. However, HPV 58 is considered a rare type, and not commonly reported in most of Europe, both Americas and Asian countries (14). Yet in Africa, this type was observed e.g. east, central, west and South Africa [14]. This finding could refer to an international difference in HPV distribution. On the other hand our results were partially in agreement with Clifford *et al*., who described the overall predominant role for HPV 16 and HPV 18. We only identified HPV 16 and could not isolate HPV type 18 often the most common high risk type [15]. All the high risk HPV genotypes identified were associated with moderate dyskaryosis and CIN2. Interestingly, our findings may be cautiously compared with the results of other African countries. Firstly, a higher prevalence of HPV has been reported among women with HIV [16] and we have recently reported low prevalence of HIV among pregnant Sudanese women [17]. Secondly there are many social and behavioral differences between different settings e.g. male circumcision -which has been reported to have low prevalence for HPV-is one of the religious practices in central Sudan [18].

## Conclusion

In summary this preliminary reported concerning HPV types in Sudan will need to be extended to larger studies in the future. Information about HPV types in Sudan will be of great value to help the national health authority and decision makers to introduce HPV vaccine in Sudan. Further studies are also needed to identify the association between HPV infection and cervical cancer.

## Competing interests

The authors declare that they have no competing interests.

## Authors' contributions

MMS and IA designed the study and carried out statistical analyses. MMS and MS carried out the cytological and histopathological investigation participated in the statistical analysis and procedures. KH HPV supervised detection and Identification. KT participated in the clinical work. All the authors read and approved the final version.
